# Phylogeography of Bone Metastasis: Clonal Evolution, Skeletal Niche Adaptation, and Clinical Implications

**DOI:** 10.3390/ijms27156805

**Published:** 2026-07-29

**Authors:** Samaa Alotab, Rasha Alissa, Mariam Zainab, Labibah Labib Khamies, Khalid Said Mohammad

**Affiliations:** 1 Department of Biochemistry, College of Medicine, Alfaisal University, Riyadh 11533, Saudi Arabia; salotab01@alfaisal.edu (S.A.); mzainab01@alfaisal.edu (M.Z.); 2 Department of Anatomy, College of Medicine, Alfaisal University, Riyadh 11533, Saudi Arabia; ralissa@alfaisal.edu; 3 Department of Clinical Skills, College of Medicine, Alfaisal University, Riyadh 11533, Saudi Arabia; lalswelhi@alfaisal.edu

**Keywords:** bone metastasis, phylogeography, clonal evolution, disseminated tumor cells, tumor dormancy, bone microenvironment

## Abstract

Bone metastasis is often treated clinically as a late complication of advanced cancer, yet accumulating evidence indicates that it is also a spatial evolutionary process shaped by clonal selection, niche adaptation, dormancy, and reseeding. This review examines BoM through a phylogeographic framework that links tumor ancestry with anatomical location and time. We discuss how heterogeneous primary tumors generate bone-tropic subclones, how circulating tumor cells pass through dissemination bottlenecks, and how disseminated tumor cells enter perivascular and endosteal niches that either maintain dormancy or support early micrometastatic outgrowth. We then compare clonal architectures across breast, prostate, lung, and renal cell carcinomas, emphasizing both lineage-specific programs and convergent bone-adaptive states, including osteomimicry, immune evasion, metabolic plasticity, and epigenetic remodeling. Methodological platforms such as multiregion sequencing, single-cell and spatial transcriptomics, lineage tracing, and liquid biopsy are evaluated with attention to the technical limitations imposed by mineralized tissue. Finally, we consider how bone lesions may function as reservoirs for secondary dissemination and how evolutionary thinking could improve biomarker development, dormancy prediction, trial design, and therapy selection. Viewing BoM as an evolving ecosystem may help shift the field from reactive skeletal management toward earlier, biology-informed intervention.

## 1. Introduction

### 1.1. Clinical Importance of Bone Metastasis

The clinical burden of bone metastasis (BoM) in solid tumors is immense, with incidence rates among metastatic cases reaching 88.74% in prostate cancer (PCa) and 53.71% in breast cancer (BCa) [1]. In patients with EGFR-mutated non-small cell lung cancer (NSCLC), the incidence of bone involvement at diagnosis is as high as 54% [2]. Lung cancer, more broadly, exhibits the highest prevalence of BoM (18.05%) at initial diagnosis across the entire patient cohorts [3]. These metastases frequently result in skeletal-related events (SREs), which include pathologic fractures, spinal cord compression, hypercalcemia, and the necessity for bone-targeted radiation or surgery [2,3]. Approximately 39% of patients with BoM experience at least one such event, leading to significant morbidity [2]. The mortality impact is stark, with roughly 350,000 deaths annually in the United States attributed to BoM disease [1]. Bone-dominant disease often follows a long clinical course, particularly in subgroups like EGFR-mutated NSCLC, where post-metastatic survival can be prolonged [2]. This clinical behavior differs from visceral metastases, as bone-only involvement may require distinct treatment strategies and carries a different prognosis compared to cases with concomitant extraosseous spread [1,3].

### 1.2. Why an Evolutionary Framework Is Needed

Traditional “seed and soil” models are limited, necessitating an evolutionary framework that views BoM as a dynamic process of clonal diversification, selection, and adaptation [4,5]. BoM involves microevolution, defined as the accumulation of genetic and epigenetic variations that lead to the formation of sub-clonal populations with distinct biological features [5]. The BME actively shapes these evolutionary trajectories, invigorating metastatic seeds through significant epigenomic reprogramming [4]. Selection is a critical component, as the initial wave of metastasis imposes a selective pressure where only certain clones capable of surviving in the foreign bone milieu can successfully colonize it [4,5]. Within this niche, ecological interactions determine cell fate; for instance, endothelial cells may maintain cancer cell dormancy, whereas osteogenic cells promote proliferation and progression toward micrometastases [4]. Transitions from indolent to aggressive behaviors in bone lesions are often underpinned by shifts in these specific microenvironmental niches [4]. Crucially, bone lesions are not merely terminal destinations but can act as sources of further dissemination, with phylogenetic evidence showing parent–child relationships in which bone clones seed distant organs [4]. Adaptation to the bone niche is further characterized by metabolic reprogramming, such as increased fatty acid metabolism in specific epithelial cell subtypes [5].

### 1.3. What “Phylogeography” Means in Cancer

Phylogeography in oncology is defined as the reconstruction of tumor lineages across anatomical sites over time to recount the history and chronology of metastatic dissemination [6,7]. This approach allows researchers to reconstruct ancestral relationships in space and time by tracing multiple cancer-specific somatic mutations simultaneously [6]. While it overlaps with tumor phylogenetics, which uses sequence variation to infer ancestry, phylogeography specifically resolves the rate, order, and directionality of metastatic events [7]. It integrates with spatial oncology by providing quantitative maps of subclone composition across tissue sections to study clonal growth patterns [6]. However, it is distinct in that genetic progression, representing the historical order of events, does not always translate directly to transitions in histological state [6]. Phylogeography also addresses organotropism and metastatic seeding analysis by describing topologies such as reseeding, seeding cascades, and parallel seeding [7]. These analyses help identify intrinsic differences in tissue tropism and clarify the complex “parent-child” routes cancer takes as it spreads throughout the body [4,7].

### 1.4. Scope of the Review

This review examines tumor phylogeny and spatial dissemination in BoM through an integrated evolutionary framework, tracing the process from primary tumor subclonal selection and circulating tumor cell (CTCs) bottlenecks through early skeletal niche colonization, dormancy, and reactivation. The bone microenvironment (BME) is characterized as an active, evolutionary ecosystem that imposes convergent selective pressures shaping clonal architecture across breast, prostate, lung, and renal cell carcinomas. Methodological frameworks enabling phylogeographic reconstruction, including multiregion sequencing, single-cell approaches, spatial transcriptomics, lineage tracing, and liquid biopsy, are critically evaluated with attention to the technical constraints specific to mineralized tissue. The concluding sections address immune evolution within the bone metastatic microenvironment, its contribution to immunotherapy resistance, and the translational implications of this evolutionary framework for biomarker development, dormancy prediction, evolution-informed therapy, and bone-specific clinical trial design.

To maintain a phylogeographic focus, this review is organized around four linked questions: first, which tumor clones acquire skeletal fitness; second, when and through which routes these clones disseminate to bone; third, how the BME selects, suppresses, or reactivates disseminated tumor cells; and fourth, how these evolutionary trajectories can be reconstructed and therapeutically exploited. This structure allows bone metastasis to be examined not only as a biological endpoint, but as a spatially and temporally evolving disease state.

## 2. Conceptual Framework: Bone Metastasis as a Spatial Evolutionary Process

### 2.1. Primary Tumor Heterogeneity as the Substrate for Dissemination

Intratumoral heterogeneity is a fundamental driver of cancer progression, as primary tumors consist of diverse subpopulations rather than uniform cell types [8]. Metastatic competence is not uniformly distributed across these populations; only a specific subset of cells possesses the traits necessary to spread to distant organs [8]. Specifically, bone-colonizing capability likely emerges as a rare trait from pre-existing or evolving subclones, as only a small percentage of cells in a primary tumor harbor the ability to achieve bone-specific colonization [9]. High-resolution phylogenetic tracing confirms that metastases consistently derive from spatially localized, expanding subclones of the primary tumor [10]. These rare subclonal expansions utilize distinct transcriptional programs that enable both local tumor progression and successful seeding of distant tissues [10].

### 2.2. Linear Progression Versus Parallel Progression

The linear model of metastasis predicts that tumor cells accumulate significant genetic and epigenetic alterations within the primary tumor before spreading to secondary sites [11]. In contrast, the parallel model suggests that dissemination occurs early in tumor evolution, with less advanced tumor cells gaining metastatic capabilities independently at ectopic sites [11]. These models have profound clinical implications, as a linear route suggests early primary tumor eradication can prevent spread, while parallel progression emphasizes the need for early systemic therapy [11].

Bone-dominant disease harbors substantial inter-lesional clonal heterogeneity through both monophyletic and polyphyletic seeding, supporting mixed rather than purely linear evolutionary trajectories in skeletal disease [12]. Early dissemination to the bone allows cells to evolve separately from the primary tumor, a process influenced by the unique selective pressures of the BME [8]. Furthermore, the risk of late-onset BoM can persist for years or decades, reflecting a prolonged state of parallel evolution during cellular latency [13]. The exact dominance of linear versus parallel routes remains unresolved in many cancers because metastatic evolution is an intricately complex process [11]. Genomic and phylogenetic analyses demonstrate that metastatic progression patterns vary substantially by malignancy: colorectal, lung, and pancreatic cancers follow predominantly linear trajectories, whereas they exhibit early dissemination from microscopic primary lesions with lower driver concordance (59%), partly reflecting treatment-driven selection of private resistance mutations that mimic parallel divergence; ultimately, inferred routes are shaped by sampling density, spatial tumor structure, and therapeutic intervention [14]. The interplay of repeated waves of seeding, metastasis-to-metastasis spread, and periods of dormancy makes reconstructing these evolutionary paths a significant challenge [11].

### 2.3. Monoclonal Versus Polyclonal Seeding

**Figure 1:**
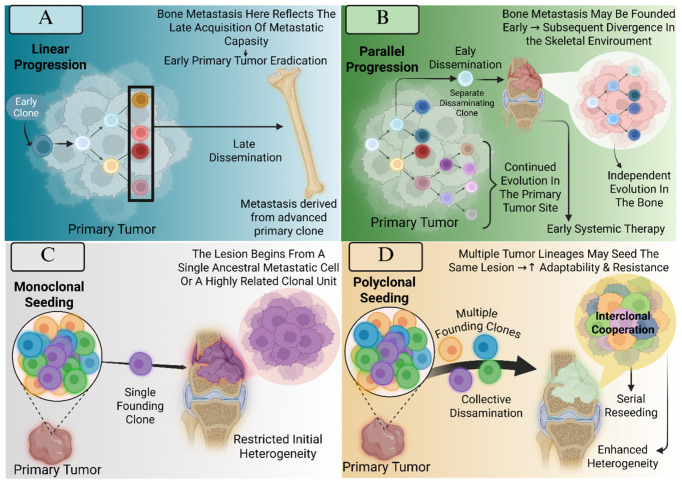
Evolutionary models of skeletal dissemination and metastatic lesion founding. (**A**) In the linear progression model, metastatic capacity is acquired relatively late during primary tumor evolution, and bone metastasis arises from an advanced primary-tumor clone after the accumulation of additional genetic, epigenetic, or phenotypic traits. This model implies that early eradication of the primary tumor may prevent subsequent dissemination if metastatic competence has not yet emerged. (**B**) In the parallel progression model, tumor cells disseminate early, establish independent disseminating clones, and continue evolving separately within the primary tumor and skeletal microenvironment. This model is particularly relevant to bone metastasis because dormant disseminated tumor cells may persist and diverge in bone before overt lesions become clinically detectable. (**C**) Monoclonal seeding refers to the establishment of a metastatic lesion by a single founding clone, or by a highly related clonal unit, producing relatively restricted initial heterogeneity. (**D**) Polyclonal seeding occurs when multiple tumor lineages collectively seed the same skeletal lesion. Interclonal cooperation, competition, serial reseeding, and local niche selection can increase intralesional heterogeneity, adaptability, and therapeutic resistance. These models are not mutually exclusive and may coexist within the same patient across different lesions or stages of disease progression. Created in BioRender. Mohammad, K. (2026) https://BioRender.com/7i5pkwu.

While some untreated metastases have been described as monoclonal, evidence from experimental models and multi-region analyses indicates that many metastatic lesions, including skeletal lesions in selected settings, can be multiclonal, founded by multiple clusters of independent clones [4]. This polyclonal seeding has significant implications for treatment resistance, as heterogeneous lesions are more likely to contain subclones capable of surviving standard therapies [8]. Within these lesions, clones can engage in interactions such as cooperation or competition, which fundamentally influence the fitness and growth of the metastasis [8]. The complexity of these lesions is further increased by reseeding mechanisms, in which cancer cells enter the circulation to seed distant sites or return to the original tumor [4]. Emerging clinical and experimental evidence further suggests that bone itself may act as an intermediate transfer station, in which cancer cells that initially seed the skeleton are subsequently reinvigorated by the BME to undergo secondary dissemination to visceral organs such as the lung, liver, and brain [15]. Phylogenetic analyses indicate that bone lesions frequently arise from later subclonal expansions rather than from early dissemination, and that multiple BoM within a patient are often clonally distinct, implying repeated seeding events from soft-tissue reservoirs rather than intraosseous spread [16,17]. The major evolutionary models and lesion-founding patterns relevant to bone metastasis are summarized in Figure 1.

### 2.4. Organotropism and Why Bone Is Different

Bone is not merely a terminal destination but a unique selective landscape that defines the non-random distribution of metastases in cancers like breast and prostate cancer [9]. It is characterized by agile temporal dynamics, where up to 25% of the skeleton is replenished annually through constant remodeling [13]. This remodeling creates a growth-factor-rich microenvironment, where osteoclast-mediated bone resorption releases critical resources such as TGFβ and IGFs that actively promote cancer cell proliferation [9]. Furthermore, the bone marrow is a dynamic organ in which hematopoiesis, osteogenesis, and immune responses are precisely regulated, providing specialized microenvironments critical for controlling tumor cell colonization. Two primary niches govern this process: the endosteal niche, which regulates DTC dormancy and outgrowth, and the perivascular niche, which maintains a dormant tumor cell phenotype through paracrine signaling [18]. These dormancy-permissive microenvironments allow cells to survive in an indolent state for decades, making bones a critical reservoir for long-term evolutionary adaptation [9]. Each of these processes is examined in detail in the sections that follow.

## 3. From Primary Tumor to Bone: The Route of Metastatic Dissemination

### 3.1. Acquisition of Bone-Tropic Potential in the Primary Tumor

Bone-tropic competence can arise within the primary tumor before overt dissemination, suggesting that metastatic destination is shaped early in tumor evolution rather than only after arrival in bone [13,19]. This is supported by evidence that the primary tumor microenvironment (TME) can enrich subclones already compatible with the bone marrow milieu [19]. In this sense, bone tropism should be viewed as an early and selective phenotype rather than a purely late adaptation [13,19].

Stromal selection within the primary tumor constitutes a key pre-dissemination mechanism: in triple-negative BCa, cancer-associated fibroblasts (CAFs) limit cytokine availability, selectively enriching for clones with high Src activity and stronger PI3K-AKT survival signaling in response to CXCL12 and IGF1, both abundantly present in the bone marrow, thereby generating a subpopulation specifically fitted to survive in a marrow-like cytokine environment [19,20]. This model helps explain why BoM can be predicted from gene-expression features already present in primary tumors, a concept developed further throughout this review [19]. It also shows how organotropism may arise without bone-specific driver mutations, because the primary stroma can mimic the future metastatic site [13,19]. Thus, similarity between the cytokine composition of the primary tumor and bone marrow can preselect cells with skeletal fitness before dissemination occurs [19]. PREX1 drives spontaneous primary-to-bone dissemination in ER+, with PREX1 knockdown significantly reducing skeletal spread from orthotopic primary sites, demonstrating that bone tropism can be intercepted at the dissemination step rather than only at colonization [21]. Further tumor-intrinsic drivers of bone-tropic dissemination are discussed in the following sections.

### 3.2. Circulating Tumor Cells and Dissemination Bottlenecks

After intravasation, CTCs enter a highly selective phase in which fewer than 0.02% form secondary tumors. Three principal barriers: hemodynamic shear stress, anoikis, and immune surveillance, collectively constitute the evolutionary bottleneck that filters for a rare subset with metastasis-initiating competence [22,23,24,25].

One barrier is Hemodynamic shear stress, which selects for a small CTC subpopulation with metastasis-initiating traits, including stemness, plasticity, and invasive capacity, through CXCR4-PI3K-AKT signaling, thereby establishing circulatory survival as an actively selective property rather than a passive one [23].

A second barrier is anoikis. Because CTCs are detached from their native matrix, they must resist anoikis to survive in suspension [22]. In suspended BCa, restoring anoikis sensitivity increased apoptosis and reduced experimental metastasis, showing that anoikis resistance is essential for dissemination [25].

A third barrier is immune attack. CTCs face natural killer cell surveillance, and platelet coating can protect them from being killed and promote metastasis [24]. In this mechanism, platelet contact induces CD155, which suppresses NK-cell cytotoxicity through TIGIT; blocking this interaction restores immune control and reduces metastasis [24]. Whether TIGIT blockade exerts a dual effect, simultaneously restoring circulatory NK surveillance and enhancing cytotoxic T cell infiltration within the bone metastatic microenvironment, as discussed later in this review, represents a compelling open question for future investigation.

### 3.3. Disseminated Tumor Cells in Bone Marrow

DTCs in the bone marrow represent the earliest clinically meaningful seeds of future metastatic disease, detectable even in patients without radiologically evident metastases, and widely regarded as the latent reservoir from which bone relapse and visceral spread emerge [26,27,28,29]. Their clinical significance is reinforced by consistent associations with relapse risk, poor survival, and overt metastatic progression, particularly in patients who can relapse after complete primary tumor removal, a phenomenon that has been linked to DTC survival in marrow niches [26,27]. The persistence of these cells during follow-up may suggest minimal residual disease that has escaped eradication by the initial therapy and remains capable of reactivation later [26]. Bone marrow is uniquely suited as a DTC sanctuary: its vascular, cytokine-rich microenvironment supports tumor cell survival while simultaneously promoting quiescence through immune, osteoblastic, and perivascular niche interactions, E-selectin adhesion, and SDF1-related chemokine signaling [26,28]. This sanctuary function helps explain why marrow DTCs are clinically relevant long before frank BoM became visible on conventional imaging [26,29]. At the same time, bone marrow DTCs are notoriously difficult to detect and study. One reason is purely technical: they are extremely rare, often occurring at very low frequencies within a complex background of hematopoietic and stromal cells [27,28]. Another reason is anatomical and procedural, as bone marrow aspirations are invasive and uncomfortable, limiting routine serial sampling compared with peripheral blood analysis [26,28]. A further obstacle is that bone marrow itself is a biologically heterogeneous tissue rich in immune cells, extracellular components, and autofluorescent material, which complicates the enrichment, staining, and interpretation of rare tumor-like cells [28]. Detection is also hindered by the biology of the DTCs themselves. These cells are often dormant or slowly cycling, which makes them less conspicuous to methods that depend on high metabolic activity or overt lesion formation [26,29]. Because conventional imaging modalities such as bone scans, CT, MRI, or PET-CT are designed to detect structural or metabolic consequences of tumor growth rather than isolated dormant cells, marrow DTCs often remain below the radiologic detection threshold [29]. This disconnect between biologically significant dissemination and clinically visible metastasis is precisely why DTCs can mark danger well before standard diagnostic tools confirm skeletal disease [28,29].

Traditional DTC detection methods, cytokeratin immunocytochemistry and RT-PCR, demonstrated occult dissemination but lacked sufficient resolution to distinguish true tumor-derived cells from morphologically similar nonmalignant marrow cells. Single-cell genomic studies directly confirmed this ambiguity: bone marrow aspirates contained genuine DTCs alongside genomically normal and tumor-unrelated aberrant cells, indicating earlier methods likely misclassified or overestimated the true metastatic population [27,28]. The single-cell and spatial platforms that resolve this limitation, enabling phylogenetic reconstruction of DTC origins, characterization of dormancy-associated transcriptional programs, and identification of therapy-evasive subpopulations, are addressed in Section 9 [27,28]. DTCs additionally differ molecularly from the primary tumor in receptor status, EMT phenotype, stem-like behavior, and osteomimicry, conferring resistance to therapies designed against the primary clone [26,28]. Clinically, SE-iFISH detected DTCs in patients without radiographic BoM, and higher CK18-positive DTC burden independently associated with shorter overall survival in, reinforcing that marrow DTCs can precede and predict overt metastatic progression [29].

### 3.4. Early Colonization of Skeletal Niches

Upon extravasation through the permeable bone marrow endothelium, DTCs first encounter the perivascular niche, a functionally plastic compartment supporting survival, quiescence, or proliferation depending on local signals [30]. This niche is shared with hematopoietic stem cells, and DTCs can compete for HSC occupancy, though in colorectal cancer models, most perivascular DTCs do not directly co-localize with HSC-enriched positions, indicating niche overlap is possible but not obligatory [31,32]. Critically, chemoresistance in the perivascular niche is not merely a byproduct of quiescence but a direct consequence of endothelial protection: DTCs receive survival signals through integrin-mediated interactions with endothelial VWF and VCAM-1, and blocking these interactions restores sensitivity to doxorubicin and paclitaxel regardless of proliferative status, demonstrating that niche attachment determines therapeutic vulnerability from the outset of metastatic residence [31].

As colonization progresses, DTCs engage the endosteal niche, where heterotypic adherens junctions with MSCs, preosteoblasts, and osteoblasts activate mTOR signaling to drive early-stage bone colonization. These early micrometastases are surrounded by ALP-positive and collagen I-positive osteogenic cells rather than osteoclasts, establishing that initial skeletal colonization occurs within an osteogenesis-rich environment that is biologically distinct from the later osteolytic vicious cycle. Cancer cells further exploit cadherin-based junctions and calcium transfer within this niche to gain a proliferative advantage, confirming that niche contact is actively instructive rather than merely permissive [33]. The transition from perivascular residence to endosteal engagement thus represents the critical progression from solitary dormant DTCs to early micrometastatic lesions, a phase that precedes, and should not be conflated with, overt bone destruction [30,33]. Key insights from this section are summarized in Box 1.

Box 1 **Which tumor clones acquire skeletal fitness?**

***Key insights:***

**Figure 2:**
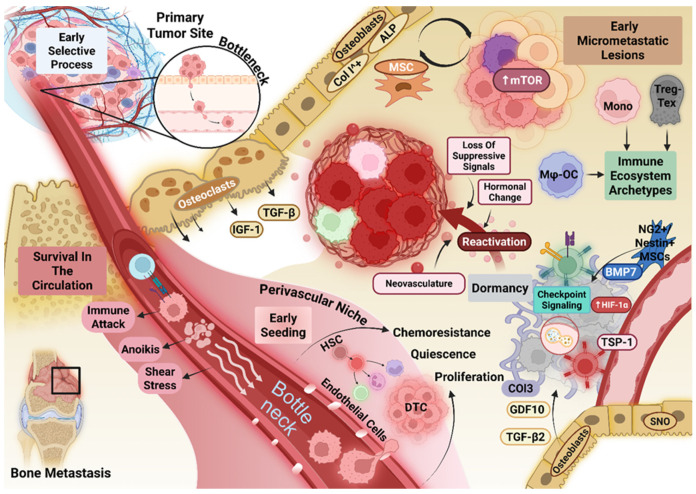
Dissemination bottlenecks, skeletal niche selection, dormancy, and reactivation in bone metastasis. The schematic illustrates the transition from primary-tumor selection to clinically relevant bone colonization. Within the primary tumor, early selective pressures enrich rare subclones with dissemination and bone-adaptive potential. After intravasation, circulating tumor cells (CTCs) pass through a stringent evolutionary bottleneck shaped by shear stress, anoikis, and immune attack, allowing only a small subset to survive in circulation. Following extravasation into bone marrow, disseminated tumor cells (DTCs) encounter specialized perivascular and endosteal/osteogenic niches. Perivascular endothelial interactions can support survival, quiescence, and chemoresistance, whereas engagement with osteoblasts, mesenchymal stromal cells (MSCs), and ALP-positive/collagen I-positive osteogenic cells can promote early micrometastatic growth and mTOR activation. Bone remodeling further shapes this process: osteoclast-mediated resorption releases matrix-derived factors such as TGF-β and IGF-1, while stable vascular, osteoblastic, and stromal signals including TSP-1, GDF10, TGF-β2, BMP7, and NG2+/Nestin+ MSC-derived cues contribute to dormancy maintenance. Reactivation may occur when suppressive niche signals are lost or overridden by hormonal changes, osteoclast activation, neovascular remodeling, hypoxia, checkpoint-associated immune escape, or immune ecosystem remodeling. The figure emphasizes that bone metastasis is not a single-step event but a sequence of selection, survival, niche adaptation, dormancy, and possible reawakening. Abbreviations: CTC, circulating tumor cell; DTC, disseminated tumor cell; HSC, hematopoietic stem cell; MSC, mesenchymal stromal/stem cell; Mφ-OC, macrophage–osteoclast immune archetype; Treg-Tex, regulatory/exhausted T-cell archetype; TSP-1, thrombospondin-1. Created in BioRender. Mohammad, K. (2026) https://BioRender.com/3y2ljpp.

- Main finding: Bone tropism can be established early in tumor evolution, and dissemination to bone unfolds as a multistep phylogeographic process involving primary-tumor priming, circulating tumor cell bottlenecks, marrow entry, and early colonization of perivascular and endosteal niches. Refer to Figure 2.
- Significance: This reframes skeletal metastasis as an evolutionary trajectory rather than a single metastatic event and highlights multiple opportunities for interception before overt bone destruction develops.
- Open question: Which transition is the most actionable clinically: suppressing pre-dissemination priming, blocking survival in circulation, or disrupting early niche adaptation in bone marrow?

## 4. Bone as an Evolutionary Ecosystem

### 4.1. The Bone Microenvironment as a Selective Filter

The bone marrow functions as a rigorous evolutionary ecosystem in which stromal, vascular, and immune components act as active selective agents rather than passive scaffolds, a process that begins even before dissemination, as primary tumor stroma can predetermine bone-specific metastatic tropism [19]. Upon arrival, DTCs undergo Darwinian selection within the perivascular niche, where endothelial interactions confer chemoresistance [31,33], and through an immunosuppressive landscape enriched in regulatory T cells that further shield resident tumor cells from immune clearance. As colonization advances, osteoclast-mediated bone destruction releases matrix-embedded growth factors that amplify tumor proliferation, sustaining the vicious cycle that drives metastatic progression, as discussed below [30]. Ultimately, metastatic progression in bone results from sequential filtering by the local bone ecosystem.

### 4.2. Ecological Pressures Unique to Bone

Bone constitutes a physically and biochemically distinctive metastatic habitat shaped by continuous matrix remodeling: TGF-β deposited by osteoblasts is released and activated during osteoclast-mediated resorption, directly coupling tissue destruction to pro-tumorigenic signaling [34]. Dense cortical bone physically confines the marrow, restricting vascular access to specific entry points and imposing mechanical constraints on tumor cell growth within a matrix-rich, spatially limited environment [35,36]. This structural confinement creates a constitutively hypoxic environment, activating HIF signaling in marrow-resident cells and generating spatially variable oxygen gradients across distinct vascular compartments [35,37]. Acidity generated by metabolic byproducts is detected by GPCRs on macrophage surfaces, therapy inducing immunosuppressive polarization that enables tumor immune evasion [38]. High and dynamic energy demands further impose nutrient limitations throughout the marrow, generating metabolic heterogeneity that shapes cell phenotypes and functional diversity [39]. Metastatic clones exploit these pressures by co-opting local resources, most notably the calcium reservoir of the osteogenic niche, which provides critical ions supporting proliferative expansion [33].

### 4.3. Resorptive and Osteogenic Niches as Distinct Evolutionary Filters

Bone metastases are shaped by two biologically distinct skeletal ecosystems: resorptive niches, in which osteoclast activity dominates, and osteogenic niches, in which osteoblast-lineage cells and matrix deposition are more prominent [33,34]. These environments impose different selective pressures on disseminated tumor cells, thereby filtering for subclones with distinct adaptive programs [40,41].

In resorptive lesions, bone destruction releases matrix-bound growth factors and creates a hypoxic, acidic, and metabolically stressed milieu that favors survival of stress-adapted clones [34,42]. In contrast, osteogenic lesions provide a calcium-rich, cell-contact-dependent microenvironment that promotes proliferative outgrowth, niche integration, and osteomimetic adaptation [33,40,43]. Taken together, these divergent pressures help explain why skeletal metastases are not evolutionarily uniform, but instead converge on different phenotypic states depending on whether the local lesion is predominantly resorptive or osteogenic [33,40]. Further details on how these niche-specific pressures shape clonal architecture and metastatic behavior are discussed in the following sections.

### 4.4. Niche-Driven Phenotypic Plasticity

Evidence from recent studies suggests that bone lesions do not evolve solely through the selection of fixed mutations; instead, metastatic progression is driven by an intricate interplay between clonal evolution and transcriptional plasticity [44]. While primary tumors may select for specific genetic traits, the BME functions as a dynamic orchestrator, inducing adaptive transcriptional and epigenetic changes that enable DTCs to undergo temporary or stable state changes, thereby enhancing survival and outgrowth [19,40]. In particular, the osteogenic niche induces a transient and reversible reduction in ER expression and activity in BCa bone micrometastases, a process independent of clonal selection and reflecting EZH2-mediated epigenomic reprogramming [40]. Similarly, in PCa, metastatic seeding converges on disease trajectories that involve both genomic alterations and recurrent rewiring of transcriptional programs, such as the acquisition of androgen receptor independence and the activation of alternative pathways, such as JAK-STAT and WNT, to adapt to local selective pressures [44]. These plastic state changes are further stimulated by the bone’s inherent turnover, where remodeling-activated NG2+ mesenchymal cells and the remodeling of the vascular endothelium create specialized niches that regulate the balance between dormancy and proliferative reactivation [45,46]. Ultimately, the survival of DTCs in the bone ecosystem is not merely a product of their initial genetic makeup but rather the result of a continuous co-evolution between tumor cells and their microenvironment, in which niche-driven epigenetic adaptation provides the flexibility necessary for successful colonization and therapy resistance [40,44]. Further cancer type-specific mechanisms are detailed in Section 6.

### 4.5. Convergent Evolution in Bone Metastasis

Convergent evolution in BoM demonstrates that disparate primary tumors frequently arrive at similar bone-adapted phenotypes despite distinct mutational profiles, a phenomenon driven by the finite and reproducible selective pressures imposed by the skeletal niche [47]. This convergence operates across multiple biological dimensions simultaneously. At the immune ecosystem level, single-cell profiling has identified three reproducible archetypes, Mφ-OC (macrophage-osteoclast), Mono (monocyte), and Treg-Tex (regulatory and exhausted T cell), that do not strictly coincide with the tissue of origin, demonstrating ecological convergence across cancer types [47]. At the stromal level, NG2+ mesenchymal cells participate in metastatic initiation across breast, prostate, colon, and lung cancers [45], while mammary tumor cells remodel bone marrow vasculature through endothelial sprouting and compression patterns remarkably similar to those in hematological malignancies [46]. At the molecular level, Osteomimicry, defined by the expression of bone-associated proteins such as osteopontin, BMPs, and cadherin-11, is a recurrent feature that facilitates niche integration in BoM across tumor types [43,48], while metabolic adaptation to hypoxia and nutrient constraints, alongside dormancy-proliferation transitions mediated by p38 MAPK downregulation, CDK8 activation, and WNT signaling, are broadly conserved [41,42]. BME-induced epigenomic reprogramming, orchestrated by HGF and TGFβ1, further drives reversible mesenchymal–epithelial shifts that enable metastatic seeds to disseminate independently of initial clonal selection [40,49]. The cancer type-specific patterns and molecular drivers underlying these convergent principles are examined in detail in Section 6. Key insights from this section are summarized in Box 2.

Box 2 **How does the BME select, suppress, or reactivate DTCs?**

***Key insights:***

- Main finding: The BME acts as a hierarchical evolutionary filter in which distinct niches (perivascular, endosteal, resorptive, and osteogenic) impose selective pressures that determine whether DTCs survive, are eliminated, or adapt through phenotypic reprogramming. Refer to Figure 2.
- Significance: This positions the bone not merely as a regulator of dormancy, but as an active ecosystem that drives metastatic fitness through niche-specific selection, ecological constraints, and convergent adaptation across tumor types.
- Open question: Which niche-specific selective pressure is most critical to target therapeutically to interrupt early/late metastatic adaptation within the bone ecosystem?

## 5. Dormancy, Latency, and Reactivation in Bone Metastasis

### 5.1. Evidence for Dormant DTCs in Bone

Bone metastatic dormancy represents a major clinical challenge: DTCs can persist in a cell-cycle-arrested state within endosteal and perivascular niches for decades, protected from both cytotoxic therapy and immune surveillance [41,50]. In ER+ BCa, where bone is the predominant metastatic site, approximately one-third of patients harbor bone marrow DTCs at diagnosis, yet roughly half never develop overt macrometastasis, and clinical relapses can occur up to 25 years after primary treatment [50]. In PCa, early dissemination followed by prolonged dormancy accounts for the 20–45% relapse rate observed despite apparently curative prostatectomy, with BoM documented decades after surgery [51,52]. Because dormant DTCs are quiescent, they are inherently resistant to therapies targeting proliferating cells, positioning dormancy as the central unresolved obstacle to preventing late bone metastatic relapse [41,52].

### 5.2. Mechanisms Sustaining Dormancy

Bone marrow DTC dormancy is actively maintained by specialized niches, especially endosteal and perivascular compartments that provide quiescence signals [53]. DTCs often engage in the endosteal and/or perivascular niches upon arrival in the bone marrow, where they can be maintained in a dormant state for extended periods [41]. These niches are required for dormant cells to persist over long durations, offering protection from both drug treatments and immune surveillance [41].

#### 5.2.1. Osteoblastic/Endosteal Dormancy Signals

Differentiated osteoblasts secrete pro-dormancy factors such as GDF10 and TGF-β2, which activate the TGFβRIII-p38MAPK-phospho(S249/T252)-RB signaling pathway in cancer cells [52]. This pathway leads to the phosphorylation of the Retinoblastoma (RB) protein at specific N-terminal sites, stabilizing the cell cycle inhibitor p27 and blocking G1 cell cycle progression [52]. Furthermore, spindle-shaped N-cadherin+ osteoblasts (SNOs) can reinforce dormancy through Jagged-1/Notch signaling or the secretion of Wnt5a, which inhibits proliferative Wnt/β-catenin signaling [54]. Direct physical contact with osteoblasts also seems required for quiescence induction, involving the inactivation and nuclear translocation of focal adhesion kinase (FAK) [51].

#### 5.2.2. Perivascular Dormancy Signals

In the perivascular niche, stable, mature microvasculature is a critical regulator of tumor cell dormancy [55]. Endothelial cells in these stable regions secrete and deposit thrombospondin-1 (TSP-1) into the basement membrane, which functions as an angiocrine tumor suppressor, maintaining sustained quiescence [55]. This environment is further supported by perivascular NG2+ Nestin+ MSCs, which promote dormancy by secreting TGF-β2 and BMP7 [41].

#### 5.2.3. Immune and ECM-Mediated Dormancy

Immune equilibrium and structural matrix components further enforce the dormant state [54]. Silencing of IRF7 promotes BoM through immune escape, showing that loss of interferon-linked immune control can weaken dormancy [56]. Simultaneously, in some contexts, dormant or slow-cycling DTCs can adopt immune-evasive phenotypes, including altered antigen presentation and increased checkpoint ligand expression, such as PD-L1 [54]. Structurally, dormant cancer cells themselves assemble a type III collagen-enriched ECM niche that maintains quiescence through DDR1-STAT1 signaling; DDR1 knockdown in established dormancy models and in bone marrow-derived cancer cell clones in vivo was sufficient to drive outgrowth of previously dormant cells, confirming that the collagen III-DDR1-STAT1 axis is not merely permissive but actively required for skeletal dormancy maintenance [57].

#### 5.2.4. Metabolic Dormancy

Metabolic adaptation is essential for the long-term persistence of dormant cells [41]. Dormant cells often shift their metabolism toward oxidative phosphorylation (OXPHOS) and rely on autophagy as a protective mechanism to survive metabolic and oxidative stress [41]. In certain models, osteoblast-induced dormancy is characterized by a significant suppression of mitochondrial protein-coding genes and a reduction in mitochondrial DNA copy number [51]. Additionally, the constitutively hypoxic nature of the bone marrow further reinforces DTC quiescence through HIF-1α stabilization, which transcriptionally orchestrates stemness maintenance, epithelial–mesenchymal transition, immune evasion, and metabolic reprogramming, collectively sustaining a therapy-refractory dormant phenotype within the skeletal niche [41,58].

### 5.3. Evolution During Dormancy

Dormancy should be viewed as a dynamic “on-off” process rather than a state of complete biological inactivity [41]. While clinically silent, bone marrow-resident DTCs continue to adapt through nongenetic cell-state transitions driven by epigenetic reprogramming [54,59]. In ER+ BCa, therapy-induced dormancy is associated with global heterochromatinization, including H3K9me2, H3K27me3, and H4K20me3 modifications, without recurrent genetic alterations, demonstrating that dormant cells can evolve through epigenetic mechanisms independently of mutational change [59].

This dormant epigenome is unstable; progressive erosion of repressive histone marks permits low-level phenotypic drift and sporadic cell cycle re-entry, evidenced by “failed awakenings” in which cells transiently proliferate under therapeutic stress before returning to arrest or being partially eliminated [59]. These epigenetic shifts regulate the balance between restrictive and permissive chromatin states and may allow low-level phenotypic drift during latency [54]. Transcriptional evolution during dormancy can further generate a stress-like cell state marked by heat-shock proteins and immediate early genes, including Fos and Jun, a program associated with enhanced tumor-seeding capacity and drug resistance [60]. The BME may further shape these evolutionary trajectories by reinforcing adaptive, stem-like phenotypes in resident DTCs [41]. Consequently, dormancy should be understood as a dynamic and heterogeneous landscape in which individual lineages can follow distinct adaptive paths before a heritable resistant phenotype emerges at reactivation [59].

### 5.4. Triggers of Metastatic Reactivation

The transition from long-term bone latency to overt metastasis is driven by clinical and biological factors that disrupt the suppressive signaling of the bone marrow niche [54]. Osteoclast activation liberates matrix-bound TGF-β1 and IGF-1, thereby activating proliferative pathways in dormant tumor cells, while endocrine changes, including estrogen or androgen deficiency, elevate IL-1, IL-6, and TNF-α, thereby enhancing RANKL expression and amplifying resorptive activity [41,54]. In PCa, DTCs can be retained in bone-associated niches, and shifts from cortical to marrow-associated compartments may further favor reactivation and progression [51]. At the niche level, the emergence of sprouting neovasculature displaces quiescence-enforcing TSP-1 signals with periostin and active TGF-β1 at neovascular tips, creating a growth-permissive micrometastatic niche [55]; simultaneously, depletion of NG2+/Nestin+ MSCs or reduction in MSC-derived TGF-β2 directly triggers dormancy escape and BoM expansion [53]. Neutrophil extracellular traps (NETs) proteolytically remodel the extracellular matrix (ECM), exposing integrin-activating epitopes that initiate cell cycle re-entry, while IRF7 silencing enables immune evasion of bone-associated surveillance, further destabilizing the dormant state [54,56]. Critically, these transitions are not unidirectional: longitudinal sampling and lineage-tracing models demonstrate that while dormant cells can reactivate into ECM-remodeling states upon osteoclast-mediated bone resorption, therapy-resistant cells can revert to dormancy after drug withdrawal [61,62]. As discussed in the preceding section, reactivation does not typically involve recurrent genetic mutations but reflects epigenetic reprogramming and transcriptional drift accumulated during prolonged latency, ensuring that emerging metastases are phenotypically diverse and potentially more therapy-resistant than the original disseminated clone [59]. The sequence from dissemination bottleneck to skeletal niche selection, dormancy, and reactivation is summarized in Figure 2. Key insights from this section are summarized in Box 3.

Box 3 **How does the BME maintain dormancy and trigger reactivation?**

***Key insights:***

- Main finding: Bone metastatic dormancy is an actively maintained and reversible state, sustained over time by stable endosteal and perivascular niche signals, in which disseminated tumor cells undergo time-dependent epigenetic and transcriptional drift with intermittent attempts at cell-cycle re-entry.
- Significance: Significance: This defines dormancy as a dynamic equilibrium rather than static quiescence, explaining how clinically silent cells can persist for decades, exhibit “failed awakenings,” and ultimately re-emerge as phenotypically diverse and therapy-resistant metastases.
- Open question: Which disruption of dormancy over time, loss of niche-derived suppressive signals, microenvironmental remodeling, or intrinsic epigenetic drift, offers the most effective strategy to durably prevent metastatic reactivation?

## 6. Clonal Architectures of Bone Metastasis Across Tumor Types

BoM are not uniform across cancers; their clonal architecture reflects a dynamic interplay between tumor-intrinsic programs and selective pressures imposed by the skeletal microenvironment [63]. Comparative analysis across tumor types reveals both conserved adaptive strategies and lineage-specific evolutionary trajectories. The methodological, phylogeographic, and evolutionary principles governing BoM across all cancer types, including multiregion sequencing, clonal heterogeneity, and lineage plasticity, are addressed in Section 3, Section 4 and Section 5 and Section 9; the following subsections focus on the cancer type-specific genomic and microenvironmental features that distinguish skeletal disease in each histology. Building on the convergent evolutionary mechanisms established in Section 4.5, this synthesis distinguishes the pan-cancer principles governing bone adaptation from the lineage-specific programs that determine how each tumor type deploys them, a distinction with direct implications for whether therapeutic strategies should be applied broadly or tailored to specific histology.

### 6.1. Breast Cancer

BCa exhibits the most extensively characterized clonal architecture in BoM [8]. It exhibits a marked tropism for bone, particularly in luminal subtypes, which account for the majority of skeletal metastases [8,64,65]. This predilection is supported by early dissemination in a subset of tumors, although the extent to which bone tropism is pre-specified versus acquired remains context-dependent [64]. The defining feature is prolonged dormancy [66]. Phylogenetic analyses and longitudinal DTC sampling demonstrate that only a subset of these dormant clones that established in the bone marrow niches described above undergo additional genetic or epigenetic changes that enable metastatic outgrowth, often involving pathways such as WNT or EGFR signaling [67,68]. Endocrine resistance drives clonal selection in BoM [69]. Activating ESR1 mutations arise under endocrine therapy and often occur in parallel across spatially distinct lesions, reflecting convergent evolution [69,70]. Consequently, bone lesions function as both reservoirs and amplifiers of endocrine-resistant subclones [69]. Niche-induced plasticity further shapes clonal architecture, as luminal tumor cells can transiently adopt basal-like or mesenchymal phenotypes after engraftment, promoting osteomimicry and survival within the BME [64,71]. Metabolically, BCa leverages de novo serine synthesis to drive osteoclastogenesis, releases lactate to fuel osteoclast-mediated bone degradation, and senses extracellular Ca^2+^ via calcium-sensitive receptors to promote proliferation [72]. Expanding on what was mentioned in Section 3, bone marrow DTC studies offer a unique window into early metastatic evolution, enabling detection of subclonal diversification before overt disease [26,73,74]. Comparative multi-site analyses, including recent metastatic BCa cancer atlases, highlight that BoM often display higher clonal diversity and distinct immune microenvironments relative to liver or lung lesions -findings further elaborated in Section 9—reinforcing the value of cross-anatomical comparisons [75]. Reflecting this immune architecture, BoM align predominantly with Mφ-OC or Mono-immune ecosystem archetypes, a distribution correlated with the high chromosomal instability burden characteristic of skeletal disease [47].

### 6.2. Prostate Cancer

PCa shows one of the strongest affinities for bone, with most patients with advanced disease developing skeletal metastases [3]. Its clonal architecture is closely linked to osteoblastic lesion biology and sustained androgen receptor (AR) signaling [76].

Tumor-bone interactions promote osteoblast activation through factors such as endothelin-1, BMPs, and WNT ligands, generating a structurally dense and biologically unique niche [76]. Within this environment, subclonal selection favors lineages adapted to osteoblastic conditions, and even within a single lesion, regional heterogeneity can reflect microanatomical differences in bone composition [77,78]. Metabolically, PCa exploits bone marrow adipocyte-derived lipids via FABP4 and adopts the Warburg phenotype through HIF-1α activation [72]. AR-driven evolution remains central throughout disease progression. Amplifications, mutations, and splice variants of AR are common in BoM, but therapeutic pressure induces diversification toward AR-independent states, including neuroendocrine differentiation associated with TP53 and RB1 loss [79,80].

Treatment-driven clonal diversification is particularly evident in bone, where serial biopsies and CTC analyses reveal polyclonal resistance mechanisms emerging over time [80,81]. Moreover, spatial heterogeneity across metastatic deposits is pronounced, with distinct bone lesions within the same patient frequently harboring non-overlapping driver alterations, consistent with parallel evolutionary trajectories shaped by local niche pressures [12,44]. Recent integrative studies emphasize that combining genomic and transcriptional profiling is essential to fully capture these dynamics, particularly the transition between AR-dependent and lineage-plastic states [82,83].

### 6.3. Lung Cancer

Lung cancer, primarily NSCLC, is a significant yet under characterized domain in the study of BoM evolution. Despite a substantial incidence of BoM in advanced NSCLC, dedicated phylogenetic and spatial analyses remain limited compared to breast and prostate cancer. Epidemiologic data indicate that 30–40% of NSCLC patients eventually develop BoM, underscoring the clinical burden [84,85]. Available data suggest that BoM often arise through branched evolutionary pathways, sometimes seeded from intermediary metastatic sites (e.g., lymph nodes or adrenal metastases) rather than directly from the primary tumor [85,86]. Osteolytic lesions predominate, driven by factors such as PTHrP and inflammatory cytokines that stimulate osteoclast activity and perpetuate a vicious cycle of bone resorption and tumor growth [85,86]. Molecularly, certain oncogenic contexts, including EGFR- and KRAS-mutant tumors, appear associated with bone dissemination [86,87]. For example, KRAS G12C mutations are more frequently associated with BoM, whereas other KRAS subtypes exhibit distinct metastatic patterns [88]. Metabolically, lung cancer -and RCC- utilize the same Ca^2+^ sensing receptor axis, establishing organ-specific metabolic crosstalk that sustains the “vicious cycle” of osteolytic or osteoblastic metastatic progression [72]. Importantly, bone-specific co-alterations and adaptive programs are not well defined. Preliminary genomic analyses suggest that BoM may exhibit elevated CDK4 amplification and other alterations distinct from those in primary tumors [86]. Unlike breast and kidney cancer, lung cancer BoM distributes evenly across all three immune ecosystem archetypes, Mφ-OC, Mono, and Treg-Tex, suggesting a less constrained immune evolutionary trajectory within the skeletal niche [47]. Critical gaps remain, including the extent of dormancy, the degree of intra-bone spatial heterogeneity, and the existence of bone-specific resistance mechanisms to targeted therapies or immunotherapy [54,89,90]. Recent imaging studies demonstrate region-specific demineralization and spatial heterogeneity in the mechanical integrity of tumor-bearing bones in lung cancer, suggesting microanatomical variation in metastatic lesions [89]. Addressing these gaps through prospective bone sampling, single-cell profiling, and longitudinal liquid biopsy, the methodological frameworks for which are detailed in Section 9, represent a critical priority for establishing lung cancer BoM as a phylogeographically characterized entity comparable to breast and prostate cancer.

### 6.4. Renal Cell Carcinoma

Renal cell carcinoma (RCC), particularly the clear cell subtype (ccRCC), offers a distinct perspective on BoM through its unique immune and stromal context [91,92]. Recent single-cell RNA sequencing studies of human ccRCC BoM demonstrate that the skeletal niche profoundly reshapes the TME compared with soft-tissue metastases [91]. Compared with soft-tissue metastases, RCC BoM exhibit reduced cytotoxic T cell infiltration and an enrichment of immunosuppressive macrophage populations, including a distinct TREM2+SPP1+ subset of tumor-associated macrophages, often coupled with osteoclast activation [91,93]. This creates a feed-forward loop in which tumor cells, immune cells, and bone-resorbing cells reinforce an immune-excluded state [92,94]. Consistent with this immune landscape, RCC BoM converge predominantly on the Treg-Tex archetype, which characteristically lacks osteoclasts, a distribution enriched in tumors with low CNA burden and reflecting a distinct immune evolutionary trajectory within the skeletal niche compared to breast and prostate cancer [47]. A distinct MSC subpopulation, transcriptionally similar to CAFs, is enriched in bone metastatic lesions and appears to contribute to bone remodeling and epithelial-to-mesenchymal transition (EMT) in tumor cells [95]. This stromal subpopulation is associated with increased activity of the RANK-RANKL pathway and decreased osteoprotegerin [96,97]. Clonally, BoM exhibit enrichment for distinct chromatin remodeling alterations, specifically SETD2 and BAP1 mutations without concurrent PBRM1 loss, indicating specialized chromatin remodeling requirements necessary for survival in the BME [95,98]. These findings underscore that, in RCC, evolutionary fitness in bone is tightly linked to immune evasion and stromal remodeling. Together, these lineage-specific patterns show that bone metastases converge on shared skeletal-adaptive outputs but reach them through distinct evolutionary routes. Table 1 summarizes the major differences in clonal architecture, timing of dissemination, dormancy, and genomic features across breast cancer, prostate cancer, NSCLC, and ccRCC, thereby reducing redundancy with the genomic discussion in Section 8.

## 7. Bone Metastases as Sources of Secondary Dissemination

### 7.1. Bone as a Reservoir of Persistent Metastatic Competence

As introduced in Section 4.5, BoM has been viewed as a terminal event in the metastatic cascade, a final sanctuary where DTCs colonize the osteoblastic or osteolytic niche, often leading to morbidity but not necessarily contributing to further systemic spread [99]. However, emerging evidence challenges this linear paradigm. Bones may instead function as a potential reservoir of viable tumor populations that may re-enter the circulation and reseed distant sites [4,100,101]. Several features support this concept. First, the bone marrow microenvironment provides unique survival signals, such as CXCL12, TGF-beta, and RANKL, which can induce a quiescent yet reversible state in DTCs, protecting them from chemotherapy and immune surveillance while retaining their proliferative and migratory potential [99,101]. Second, longitudinal genomic studies of paired bone and soft tissue metastases reveal branched evolutionary patterns, suggesting that bone-derived clones can serve as sources of subsequent metastatic diversification, consistent with the phylogeographic evidence discussed in Section 9 [4,100]. Third, preclinical models and indirect clinical evidence suggest that tumor cells isolated from bone lesions can colonize distant organs in experimental settings. Thus, rather than being a dead end, bone may represent a persistent launchpad for metastatic reseeding, a concept with direct therapeutic implications developed in Section 11 [4,102].

### 7.2. Metastasis-to-Metastasis Dissemination

The concept that metastatic lesions can seed additional metastases, so-called metastasis-to-metastasis dissemination, has gained traction in the field of metastatic evolution [4,100]. Direct proof of bone-to-viscera seeding in humans remains limited by the lack of serial lineage tracing, but genomic, ctDNA, and modeling studies are consistent with this possibility [4,100,103]. Specifically for bone, autopsy studies and ctDNA analyses have identified shared subclonal mutations between BoM and subsequent liver or lung lesions, with no evidence of those mutations in the primary tumor [103,104,105]. Furthermore, mathematical modeling of metastatic spread in PCa and BCa patients suggests that the number and distribution of secondary lesions are better explained by a model that includes metastasis-to-metastasis seeding than by a model in which each metastasis arises independently from the primary tumor [106,107]. Although definitive lineage tracing in humans is not yet feasible, the weight of circumstantial evidence suggests that bone is a plausible hub in a metastatic cross-seeding network [4,100]. This remains a major open question and a priority for future investigative models, including barcoded mouse models and longitudinal ctDNA phylogenies; further details are provided in Section 9.

### 7.3. Clinical Significance of Secondary Spread from Bone

Recognizing bone as a potential source of further dissemination fundamentally shifts the clinical meaning of BoM and has direct implications for four key areas of clinical management. For surveillance, if future longitudinal studies confirm that selected bone lesions act as reseeding hubs, stable bone-dominant disease may require different surveillance strategies than currently used, including closer integration of skeletal imaging, visceral imaging, and ctDNA-based monitoring [108,109,110]. For timing of systemic therapy, stable bone-only disease is often managed with observation or bone-directed agents alone; however, if bone reservoirs can intermittently release aggressive clones, earlier or continuous systemic therapy may be justified to suppress reseeding events, which challenges the practice of deferring second-line therapies in oligoprogressive bone disease [15,111]. For local control of bone lesions, palliative radiotherapy or surgical stabilization is typically applied for pain or fracture risk, but if a particular bone lesion is a reseeding hub, then definitive local therapy such as stereotactic body radiotherapy (SBRT), or selective embolization could reduce distant failure by eliminating a source of metastasis-to-metastasis spread, reframing local control as a potential systemic intervention [112,113].

For interpretation of oligometastatic bone disease, curative-intent local therapy to all visible lesions is increasingly common in patients presenting with few BoM, but if bone acts as a reservoir, what appears as oligometastatic may actually reflect a more disseminated but occult population capable of later reseeding, complicating patient selection for ablative therapies and suggesting that the absence of visceral disease should not be equated with limited metastatic potential [15,112]. This represents one of the field’s strongest future directions because it shifts the clinical meaning of BoM and reframes clinical decisions from managing bone disease to prevent bone-driven systemic progression. The critical unanswered question of how often bone lesions seed other lesions now require dedicated investigation using integrated genomic, radiologic, and preclinical approaches. Answering this question will determine whether bone-directed therapies should be reclassified as metastasis-prevention strategies.

## 8. Genomic, Transcriptomic, and Epigenetic Features of Bone-Adapted Metastases

### 8.1. Recurrently Enriched Genomic Alterations in Bone Metastasis

As summarized in Section 6, bone-adapted metastases across cancer types show both shared and lineage-specific clonal architectures. In this section, we focus only on the recurrent genomic alterations and signaling programs that may facilitate skeletal adaptation. Early sequencing efforts sought to identify bone-tropic driver mutations, but subsequent large-scale analyses have revealed that no single genomic alteration is either necessary or sufficient for BoM [114]. Instead, certain pathways are recurrently enriched, reflecting the selective advantage they confer in the bone milieu. Frequently altered pathways include the PI3K-AKT-mTOR axis, where PTEN loss and PIK3CA activating mutations are overrepresented in BoM from breast and prostate cancers [115,116,117]. Enhanced AKT signaling promotes survival under low-glucose conditions and upregulates the osteolytic factor PTHrP by inactivating FOXO3a [117,118,119]. TP53 dysfunction contributes to genomic instability and upregulation of NF-κB-driven cytokines (IL-6, IL-11, RANKL) that stimulate osteoclastogenesis [120,121]. TP53 mutations are frequently observed in bone-metastatic lesions from PCa and BCa, though their frequency varies by tumor type and metastatic site [120,121].

Lineage-specific alterations also contribute to bone adaptation. In PCa, TMPRSS2-ERG is associated with transcriptional reprogramming that can promote osteomimicry and interaction with the BME [122]. In BCa, ESR1 mutations and other endocrine therapy-selected alterations are associated with metastatic progression and therapy resistance, In, ESR1 mutations and other endocrine therapy-selected alterations are linked to metastatic progression and therapy resistance, but their contribution to skeletal remodeling is likely context-dependent rather than uniformly mediated through RANKL-driven osteolysis [123,124].

However, a critical caveat is that these alterations show enrichment but not specificity for BoM. TP53 mutations occur across multiple metastatic sites with varying frequencies, and PIK3CA mutations are common in primary tumors and metastases to the liver, lung, and bone [121,122]. Intrapatient heterogeneity further indicates that bone lesions within the same patient can harbor distinct driver mutations. Thus, genomic alterations act as permissive facilitators that lower the threshold for bone colonization rather than as deterministic bone-seeking alleles. The actual phenotypic outcome is co-directed by transcriptional and epigenetic states, as discussed below. The methodological platforms used to detect these alterations are discussed in Section 9.

### 8.2. Transcriptomic States Associated with Bone Colonization

Bone-adapted metastases do not conform to a single transcriptional signature but instead occupy a dynamic continuum of interconvertible states [47]. Single-cell RNA sequencing (scRNA-seq) of patient-derived BoM has identified at least six recurrent transcriptional programs that operate in parallel, often within the same lesion [47,125]. Dormancy is characterized by G0/G1 arrest, autophagy upregulation, and chemotherapy resistance via quiescence, with core markers including NR2F1, SOX9, CDKN1A (p21), and BHLHE41 [126,127,128,129,130]. Epithelial–mesenchymal plasticity (EMP) involves a partial hybrid E/M state (not full mesenchymal differentiation), marked by ZEB1, SNAI2, VIM, and retained EPCAM, which facilitates invasion through bone sinusoids while maintaining proliferative capacity [131,132,133]. Osteomimicry drives expression of bone matrix proteins such as RUNX2, SPP1 (osteopontin), IBSP (bone sialoprotein), and BGLAP (osteocalcin), allowing tumor cells to adhere to hydroxyapatite, evade anoikis, and incorporate into the bone remodeling unit [134,135]. ECM remodeling involves MMP1, MMP9, MMP13, LOX, and ADAMTS5, which degrade collagen and release TGFβ and IGF-1 from the bone matrix, thereby fueling a feed-forward growth cycle [136,137]. Inflammatory/immune-adaptive programs, marked by IL6, IL8, CXCL1, COX2, and CSF1, recruit osteoclasts and myeloid-derived suppressor cells (MDSCs), establishing an immunosuppressive niche [138,139]. Therapy resistance is driven by upregulation of drug efflux (ABCB1), anti-apoptotic factors (BCL2L1), and survival kinases (AXL, ERBB3), often co-activated with inflammatory programs [140,141]. Additionally, ACBP is selectively upregulated in bone, representing a bone-specific metabolic adaptation and independent prognostic biomarker for poor survival in breast and lung cancer [142], while MUC1- and SCGB2A2-expressing subpopulations are significantly enriched in BoM over matched primaries, implicating both in bone-homing competence [143]. Pan-cancer single-cell analyses further identified distinct stress-responsive (HSPA1A/FOS), migratory (FN1/FLT1), and proliferative (CCNB/CDC20) malignant cell states facilitating skeletal colonization, within a broader dual-phase adaptation in which primary tumors upregulate OXPHOS and lipid metabolism before dissemination, while established BoM rewire metabolic and immune signaling to match the calcium-rich, growth-factor-loaded niche [125,144]. This plasticity implies that a single biopsy snapshot is insufficient for therapeutic decision-making; rather, the transition probabilities between states, governed by niche signals such as TGFβ and hypoxia, may be more clinically relevant than the prevailing state itself.

### 8.3. Epigenetic Remodeling in Bone Metastasis

Epigenetic mechanisms drive reversible, non-genetic adaptation in BoM, explaining how genetically heterogeneous tumors converge on bone-adaptive phenotypes [63,145]. Unlike fixed mutations, chromatin modifications respond rapidly to microenvironmental cues [146]. For instance, bone-derived TGFβ induces SMAD2/3 to recruit p300, establishing super-enhancers at RUNX2 and JUNB [147]. Concurrently, EZH2-mediated H3K27me3 decreases at osteolytic promoters (MMP13, IL11) to depress these genes, while H3K9ac increases at VEGFA enhancers to support angiogenesis [148]. Beyond these promoter-level changes, bone-metastatic cells show extensive enhancer rewiring compared to primary tumors, with gained enhancers binding transcription factors (AP-1, TEAD/YAP1) that respond to mechanical strain and calcium flux; signals unique to the BME [149,150]. This rewiring is reversible, as bone-metastatic cells regain primary-tumor enhancer activity when returned to the mammary fat pads [150]. Separately, but equally critical for adaptation, low marrow oxygen (1–6% O_2_) stabilizes HIF1α, recruiting KDM3A to remove H3K9me2 at hypoxia-responsive genes (PDK1, LOX), thereby priming cells to switch from quiescent/OXPHOS to glycolytic/proliferative states upon reoxygenation [151,152]. In parallel, DNA hypermethylation silences CDKN2A and DAPK1 in BoM, whereas bivalent domains (H3K4me3/H3K27me3) at SOX9 and NANOG keep tumor cells poised for phenotypic switching between dormancy and proliferation [153,154,155]. Given this plasticity, HDAC/DNMT inhibitors can force differentiation but may reactivate dormant clones; therefore, chrono-epigenetic strategies aim to synchronize reactivation with cytotoxic vulnerability [156,157].

### 8.4. Non-Genetic Heterogeneity

**Figure 3:**
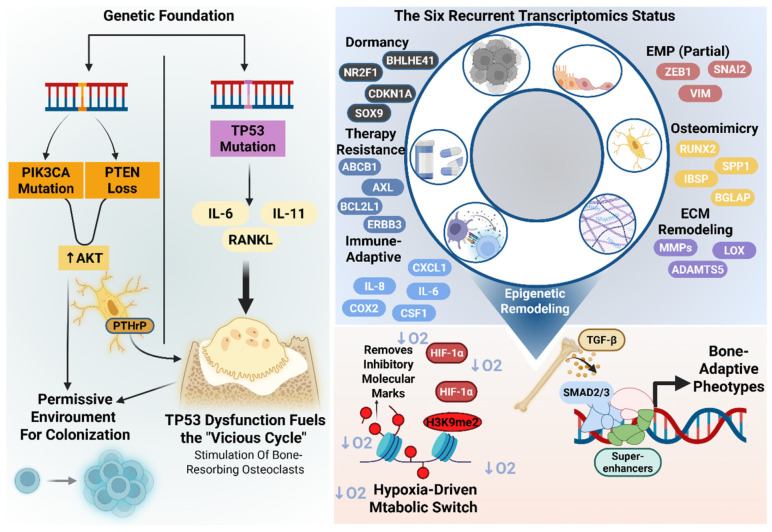
Genomic, transcriptomic, and epigenetic programs that converge on bone-adaptive metastatic phenotypes. Bone-adapted metastases arise through both fixed genomic alterations and reversible non-genetic state transitions. The left panel illustrates recurrent permissive genomic pathways implicated in skeletal colonization. PIK3CA mutation or PTEN loss can converge on AKT activation, supporting survival signaling and promoting osteolytic mediators such as PTHrP. TP53 dysfunction can amplify cytokine and osteoclastogenic programs, including IL-6, IL-11, and RANKL, thereby contributing to the tumor–bone “vicious cycle” through stimulation of bone-resorbing osteoclasts. These alterations are not deterministic bone-specific drivers but can lower the threshold for colonization in a permissive skeletal niche. The right panel summarizes recurrent transcriptomic states observed in bone metastasis, including dormancy, therapy resistance, immune-adaptive signaling, epithelial–mesenchymal plasticity, osteomimicry, and extracellular matrix remodeling. Hypoxia-driven metabolic switching, TGF-β/SMAD2/3 signaling, enhancer rewiring, and chromatin remodeling act as integrative mechanisms that allow genetically diverse clones to converge on similar bone-adaptive phenotypes. This convergence helps explain why lesions with different mutational backgrounds may display shared behaviors such as dormancy, osteoclast activation, immune evasion, and therapy resistance. Abbreviations: ECM, extracellular matrix; EMT/EMP, epithelial–mesenchymal transition/plasticity; HIF-1α, hypoxia-inducible factor-1α; PTHrP, parathyroid hormone-related protein. Created in BioRender. Mohammad, K. (2026) https://BioRender.com/z2ow39v.

BoM exhibits phenotypic convergence across genetically divergent lesions: distinct metastases or adjacent cells within the same lesion show different genotypes but similar functional behaviors [158]. For example, PIK3CA-mutated breast and PTEN-deleted prostate lesions both hyperactivate AKT, upregulating PTHrP/RANKL and leading to identical osteolytic phenotypes [159,160]. Similarly, BRCA2-mutant (promoter hypermethylation) and MYC-amplified (EZH2-mediated H3K27me3) tumors both downregulate MHC class I expression, thereby evading NK surveillance [161]. Convergence also extends to dormancy, which can be achieved via NR2F1 overexpression, p27Kip1 stabilization, or autophagy induction; all of which yield indolent, chemotherapy-refractory behavior [162,163]. This convergence has critical clinical implications: bone biopsies fail to predict therapy response in other lesions; targeted therapies show mixed responses; and phenotypic relapse occurs without new mutations because the bone niche selects for outcomes (survival, adhesion, immune evasion) rather than specific pathways, a bet-hedging strategy that ensures survival [40,164]. Finally, single-cell multi-omics (scRNA-seq + scATAC-seq) reveals that epigenetic state, not mutational status, predicts drug sensitivity, positioning chromatin modifiers as therapeutic targets if timed to avoid reactivating dormant reservoirs [165,166]. The genomic, transcriptomic, epigenetic, and non-genetic programs that converge on bone-adaptive phenotypes are summarized in Figure 3.

## 9. Methodologies for Reconstructing Bone Metastasis Phylogeny and Phylogeography

Reconstructing the evolutionary and spatial history of BoM requires an integrated methodological toolkit, as no single platform simultaneously resolves clonal identity, migration routes, functional adaptation, and temporal dynamics within the constraints of mineralized tissue [167]. These platforms are most useful when linked to specific phylogeographic questions: which clone seeded the bone, whether dissemination occurred early or late, whether lesions were founded monoclonally or polyclonally, how tumor and stromal states are spatially organized, and whether bone lesions can reseed other organs.

### 9.1. Multiregion Bulk Sequencing

A single biopsy captures approximately 40% of a tumor’s total mutations and 34.6% of its clonal populations [12,168], rendering single-site profiling insufficient for phylogeographic reconstruction. Multiregion sequencing (MRS) overcomes this by profiling spatially distinct primary and metastatic regions simultaneously, enabling subclonal architecture reconstruction and revealing that dissemination arises from specific evolutionary branches rather than a homogeneous tumor mass [167]. Rapid-autopsy profiling of 93 samples from 26 mCRPC patients confirmed that bone lesions -alongside lung metastases- carry the lowest tumor purity (the proportion of tumor cells present within a given tissue sample compared to non-cancerous cells) across all metastatic sites [12]. Though a single sample bulk DNA sequence cannot track clonal evolution from primary, the MSK-MET cohort of 25,775 patients corroborates the bone-specific genomic correlates described in previous sections, identifying AR amplification and PTEN deletion in bone-metastatic PCa, elevated chromosomal instability in HR+/HER2 ductal, and enriched PI3K pathway alterations with depleted CBFB mutations in bone-metastatic HR+/HER2 at the population scale [169].

### 9.2. DNA Sequencing

Bulk sequencing overestimates clonality and underestimates mutation rate [12]. single-cell profiling of CTCs/DTCs instead resolves the phenotypic traits of the rare clones that survive the metastatic bottleneck (Section 3.2) [167]. Phylogenetic WES/CNV analysis in breast cancer showed the dominant pattern is single-site seeding followed by metastasis-to-metastasis cascades, establishing bone as both a destination and a relay source for further spread [20] (Section 7). Consistent with this, BoM accumulates 286 somatic mutations relative to the primary tumor; with distinct private mutation sets between bilateral skeletal deposits and clinically actionable alterations, including PI3K-E545K and the acquired BRCA1-D1834H, detectable in matched circulating cell-free DNA [170].

### 9.3. RNA Sequencing

scRNA-seq enables high-resolution dissection of bone metastatic heterogeneity by constructing transcriptomic atlases from paired primary and metastatic tissues, thereby revealing distinct subpopulations, developmental trajectories, regulatory networks, and intercellular communication patterns that are inaccessible to bulk sequencing [143]. Analysis of 602,497 single cells from 124 PCa tissue samples identified neuroendocrine-like cell clusters and lineage plasticity as principal drivers of therapeutic resistance in bone, with bone-adapted cells exhibiting enhanced growth-promoting pathways and reprogrammed lipid metabolism linked to immunosuppression [5]. In BCa, scRNA-seq of rare dormant bone DTCs defined a dormancy transcriptional program (CFH, GAS6, MME, OGN, PDGFRB, MGP) that, while not individually causal, carries strong prognostic value for relapse risk [171]. Simultaneous epigenomic and transcriptomic mapping further reveals how regulatory networks are spatially organized within the bone TME, thereby driving heterogeneity [172]. Combined bulk and scRNA-seq demonstrated a luminal-to-basal/HER2 transcriptomic transition from primary tumor to BoM, with EPHA3, ROS1 upregulation, and PI3K-AKT-mTOR, angiogenesis, and EMT pathway activation across six resolved epithelial subpopulations [170], while Bulk transcriptomic profiling of PCa BoM delineated three molecular subtypes, with the androgen-responsive MetA subtype predominating in bone with a favorable prognosis [173].

### 9.4. Spatial Transcriptomics and Digital Spatial Profiling Add About Dormancy

Unlike bulk sequencing, spatially resolved transcriptomics maps cellular neighborhoods at near-single-cell resolution, uncovering cytokine gradients, immune exclusion zones, metabolic compartmentalization, and ligand-receptor interactions and, when integrated with genomic profiles, directly linking specific mutations to distinct spatial architectures within the same lesion [174]. This spatial dimension is particularly consequential in bone, where distinct lesions within the same patient frequently harbor non-overlapping driver alterations consistent with parallel evolutionary trajectories shaped by local niche pressures, dynamics fully capturable only by combining genomic and transcriptional profiling, particularly for resolving transitions between AR-dependent and lineage-plastic states [44,82,83,84]. Spatially resolved immune profiling further linked T-cell exhaustion state and spatial organization to treatment outcome [175]. For example, digital spatial profiling of BoM revealed that immunosuppressive markers, Foxp3, CD163, CD27, PD-1, and PD-L1, are selectively enriched in CD45+ cells at the endosteal surface, while proliferative markers including p38 MAPK, pan-Ras, Mek1, and phospho-Erk1/2 predominate in marrow-resident tumor cells [176].

### 9.5. Lineage Tracing and Barcoding Models

Lineage tracing, inserting heritable barcodes into the genome to track cellular progeny, has been applied in cancer to investigate clonal dynamics, treatment survival, and the origins of primary and metastatic growth in vivo [166,177]. CRISPR/Cas9 barcoding in PCa revealed that bone colonization occurred in 92.9% of metastatic cases, characterized by early polyclonal primary-to-bone seeding [177]. Beyond mapping seeding routes, barcoding has illuminated niche-induced reprogramming after bone arrival: In ER+ breast cancer, barcoding showed osteogenic niche colonization silences ER expression via EZH2-mediated reprogramming (independent of clonal selection), driving endocrine resistance that is reversible by EZH2 inhibition [40].

### 9.6. Liquid Biopsy Approaches

Single-cell CTC profiling enables genotypic and phenotypic characterization of metastasis-initiating cells, uncovering heterogeneity missed by bulk analyses and identifying targets for metastasis prevention and therapy [178]. Circulating osteocalcin-positive cells (cOC: CD15^−^CD34^−^Osteocalcin^+^) represent a sensitive early biomarker: baseline cOC levels were significantly elevated in patients developing incipient BoM versus stable disease, with a quadratic kinetic profile peaking during the early micro-metastatic osteoblastic phase before tapering with massive bone destruction [179]. In the SUCCESS A trial, persistent CTC positivity after adjuvant chemotherapy predicted bone-first recurrence, implicating chemo-resistant residual disease in bone marrow niches [180]. Because bone-only diseases are frequently RECIST-unevaluable, serial ctDNA is particularly valuable for tracking clonal evolution under therapy in this setting [181,182]. Bone marrow aspiration (BMA), a rapid and cost-effective complement to these circulating biomarkers, enables detection of metastatic non-hematopoietic cells, particularly when trephine biopsies are unsuitable for immediate evaluation. However, its sensitivity is lower than biopsy due to interpretive variability, sampling errors from focal disease, and dry taps caused by marrow desmoplasia [183].

### 9.7. Computational Frameworks

Complementing existing in vivo models with multiscale computational approaches is crucial for dissecting the complex interactions between tumor cells and the BME and for improving our understanding of metastatic progression and therapy response [183]. Integration of spatial transcriptomics with scRNA-seq through computational deconvolution has further enabled cell-type mapping within the bone marrow niche, offering a framework to dissect the spatial organization of metastatic clones, dormant DTCs, and their stromal and immune neighbors within bone lesions [184].

### 9.8. Technical Limitations Specific to Bone Metastasis

Technical limitations in BoM research arise from three interconnected levels. At the tissue processing level, decalcification compromises nucleic acid integrity: EDTA-based protocols often yield poor RNA recovery, while acid-based methods improve yield but risk mRNA over-degradation in dense bone samples if exposure is not precisely controlled; spatial transcriptomics adds further constraints through low transcriptome capture rates, small capture areas limiting statistical power, and morphological variability between sections requiring specialized computational alignment frameworks [184]. At the model level, no existing system fully recapitulates the complexity of human bone metastatic disease: 3D cultures better model tumor-microenvironment interactions than 2D systems, and ex vivo bone organ cultures preserve native cell–cell and cell–matrix contacts, yet neither captures in vivo intra-tumor heterogeneity or the full pathogenic metastatic cascade [185]. Animal models are particularly inadequate for studying dormancy and tumor homing, the precise processes most critical to understanding late relapses, compounding the already limited mechanistic insight into dormancy escape and leaving a major gap in therapeutic development [54]. At the sampling level, the invasiveness of bone marrow aspiration restricts serial monitoring; CTC-based biomarkers such as Ki-67 and M30, markers of proliferation and apoptosis, respectively, offer a promising minimally invasive alternative for capturing the dormancy, epithelial-to-mesenchymal plasticity, and stemness programs that protect minimal residual disease from systemic therapy [180,186].

An open question with direct methodological implications is whether expanding panels of bone-tropism biomarkers, capturing the stromal and transcriptional priming of bone-competent clones within the primary tumor itself, could eventually reduce the analytical burden currently placed on bone biopsy. If primary tumor sampling reliably reflects the future skeletal disease, the formidable pre-analytical constraints of decalcification, low tumor purity, and limited longitudinal accessibility that define bone biopsy could be partially circumvented without sacrificing biological resolution.

**Figure 4:**
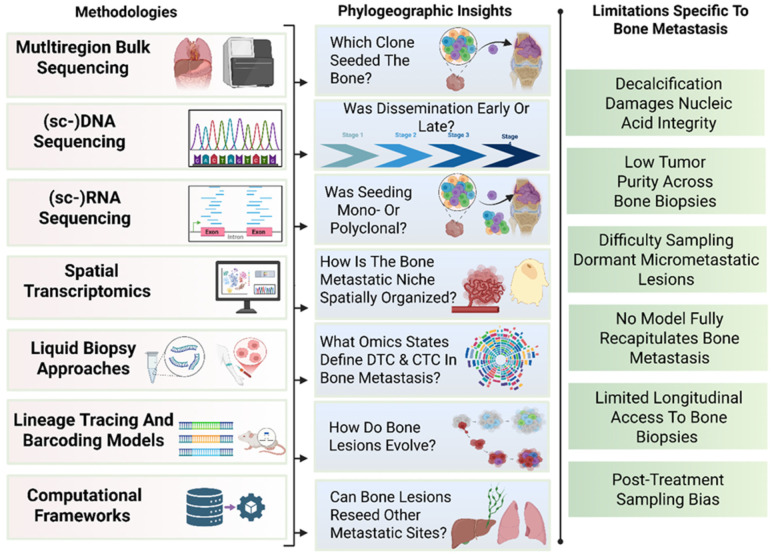
Methodological platforms for reconstructing bone metastasis phylogeny and phylogeography, and limitations specific to skeletal lesions. Reconstructing the spatial and temporal history of bone metastasis requires integration of complementary molecular and computational approaches. Multiregion bulk sequencing can define regional clonal architecture across primary and metastatic sites, while DNA sequencing identifies shared and private somatic alterations that support lineage reconstruction. RNA sequencing and single-cell transcriptomics resolve malignant-cell states, stromal remodeling, immune phenotypes, and therapy-resistant subpopulations. Spatial transcriptomics and digital spatial profiling add anatomical context by mapping tumor, immune, vascular, osteoblastic, and osteoclastic compartments within the bone metastatic niche. Liquid biopsy approaches, including circulating tumor cells, circulating tumor DNA, and bone marrow aspirate-derived cells, permit longitudinal monitoring when repeated bone biopsies are impractical. Lineage tracing and barcoding models provide experimental systems for testing dissemination timing, clonal fitness, reseeding, and polyclonal outgrowth. Together, these tools address key phylogeographic questions: which clone seeded the bone, whether dissemination occurred early or late, whether lesions were founded monoclonally or polyclonally, how the bone niche is spatially organized, what molecular states define CTCs and DTCs, how lesions evolve over time, and whether bone lesions can reseed other organs. Major limitations remain, including nucleic acid damage caused by decalcification, low tumor purity in bone biopsies, difficulty sampling dormant micrometastatic lesions, imperfect experimental models, limited longitudinal access to skeletal tissue, and incomplete resolution of immune contexture. Created in BioRender. Mohammad, K. (2026) https://BioRender.com/raypek3.

Complementary platforms used to reconstruct bone metastasis phylogeny and phylogeography, together with their bone-specific limitations, are summarized in Figure 4 and Box 4.

Box 4 **Methodological insights into bone metastasis biology.**

***Key insights:***

- MRS overcomes the limited representativeness of single-site biopsy by profiling spatially distinct regions simultaneously, resolving bone-specific subclonal architecture.
- Single cell sequencing enables direct linkage of genotype to phenotype in individual metastatic precursors supporting the possibility that bone may act as both a destination and a relay source for further metastatic spread.
- RNA-seq dissects bone metastatic heterogeneity at high resolution, uncovering distinct tumor subpopulations, dormancy-associated transcriptional programs, and lineage-plasticity states that underlie therapy resistance in bone.
- Spatial transcriptomics captures spatial differences between lesions, showing that distinct tumor deposits within the same patient may harbor different driver alterations and spatially segregated immune states shaped by local niche pressures.
- Lineage tracing and barcoding map the seeding routes of bone colonization and illuminate niche-induced reprogramming after arrival, showing the BME itself can drive phenotypic switches independent of clonal selection.
- Liquid biopsy enables early, non-invasive detection of incipient bone metastasis and minimal residual disease, ahead of what imaging can capture.

## 10. Immune Evolution in Bone Metastasis

### 10.1. Cytokine-Driven Bone Tropism, Osteoclastogenesis, and Macrophage Remodeling of the Metastatic Niche

The preceding sections describe how tumor clones adapt to skeletal stromal, vascular, and metabolic pressures. This section focuses specifically on immune evolution: how cytokine signaling, myeloid remodeling, osteoclastogenesis, and checkpoint-associated immune suppression create lesion-specific immune states that shape both clonal fitness and therapeutic response.

At the broadest level, complement signaling (C5a/C5aR1), IL-1β-PI3K/Rac-driven tumor invasiveness, osteoclast-derived lipids, exosomes, and osteoblast/RANKL-mediated crosstalk collectively orchestrate the tumor-bone communication network that initiates and sustains osteoclastic remodeling [187]. Upon arrival of cells in the BME, IL-1β is upregulated to promote a conducive metastatic niche and initiate the vicious cycle of bone destruction [18]; tumor-derived exosomal miRNAs including miR-21 and miR-183 further remodel the pre-metastatic niche to favor osteoclastogenesis [20]. Cancer type-specific osteolytic mediators amplify these effects: IL-11 drives RANKL-independent osteoclastogenesis through a JAK1/STAT3/c-Myc pathway in BCa, with its enrichment in the bone-specific BoM-1833 subclone illustrating how clonal adaptation within the skeletal niche generates increasingly aggressive osteolytic phenotypes [188]; IL-20RB enables lung cancer cells to respond to osteoclast-derived IL-19 through the same JAK1/STAT3 axis [189]; and MDA-9/Syntenin drives PCa bone tropism through a PDGF-AA/CXCL5 paracrine loop that simultaneously promotes homing, osteolytic progression, and MDSC-mediated immunosuppression [190]. PCa cells further exploit osteomimicry by expressing OCN, OPN, and ALP, thereby converting resident osteoblasts into cancer-associated osteoblasts that secrete IL-6, IL-8, IGF-1, and TGF-β to sustain tumor progression [191]. Elevated leptin receptor expression additionally enhances BCa invasiveness through SDF-1/CXCR4 axis activation [192,193].

Macrophage dynamics constitute a second major axis of niche remodeling. CD137 signaling recruits monocytes and macrophages and drives their osteoclastogenic differentiation, creating an osteolytic niche favorable for tumor colonization [194]. A distinct Ly6C+CCR2+ monocyte-derived macrophage subset marked by high CD204 and IL4R expression specifically accumulates within the bone metastatic niche to actively support metastatic outgrowth [195]. SCUBE2, an ER-regulated secretory factor in luminal, orchestrates an osteoblastic immune-suppressive niche through SHH-Hedgehog-driven MSC differentiation into collagen-rich osteoblasts that suppress NK cell cytotoxicity via LAIR1 [20,65].

### 10.2. Immunosuppressive Architecture and Single-Cell Immune Landscape of Bone Metastases

Single-cell and spatial immune profiling have revealed that BoM are not immunologically uniform, a heterogeneity with direct implications for therapeutic targeting, as introduced in Section 8.4.

At the lesion level, scRNA-seq resolved distinct immune compositions between individual bone deposits: one metastatic site was enriched for naïve T and B cells while another harbored predominantly Tregs and macrophages; critically, immune checkpoint and ligand expression within CD8+ T, NK, and Treg subsets was resolved at a resolution entirely obscured by bulk RNA-seq [170]. G-CSF promotes bone colonization by recruiting immunosuppressive myeloid cells, inducing NETs, which, as established in Section 5.4, trigger dormancy escape, and remodeling marrow vasculature in a hematopoietic cell-independent manner, while upregulation of CXCL10 and CXCL11 in bone metastasis-associated endothelial cells further shapes the inflammatory niche [46].

Collectively, bone lesions are frequently immune-cold, with cytotoxic T cell activity suppressed by MDSCs, TAMs, neutrophils, Tregs, and Th17 cells [196]. Digital spatial profiling of BoM demonstrated that blastic lesions, often considered immunologically cold, are enriched for PD-L1, B7-H4, VISTA, OX40L, IDO-1, and ICOS across tumor, macrophage, and T cell compartments, with stronger JAK-STAT activation, while osteolytic lesions exhibit greater infiltration of exhausted CD8+ T cells, TAMs, neutrophils, and Tregs with comparatively lower checkpoint molecule expression, consistent with the spatially resolved patterns discussed in Section 8.4 [197,198,199]. These lesion-type-specific immune states suggest that blastic and osteolytic BoM may require fundamentally different immunotherapeutic strategies, with blastic lesions potentially more receptive to checkpoint blockade and osteolytic lesions potentially more amenable to approaches targeting myeloid and regulatory immune populations [197,198,199].

This intra-patient immune heterogeneity, compounded by tumor evolution and the lack of clinically relevant models, and SPP1+ macrophage-CD44+ tumor cell interactions that accelerate bone destruction and worsen survival, means that while some BoM may respond to PD-1/PD-L1 blockade, others depend on androgen-response or proliferative programs that render checkpoint inhibition insufficient [125,170]. Clinically, bone lesions demonstrate significant discordance in ICI response compared to visceral lesions and independently predict worse overall survival, collectively confirming that bone metastatic disease constitutes a distinct and poorly ICI-responsive evolutionary compartment [200]. This resistance extends beyond PD-1/PD-L1 monotherapy to combined PD-1/CTLA-4 blockade, consistent with an immunosuppressive bone niche enriched in Tregs, positioning Treg depletion as a promising adjunct strategy. Notably, anti-PD-1 therapy may still confer skeletal benefit independent of tumor response by inhibiting osteoclastogenesis to limit bone destruction and pain [201]. A related emerging target is Dickkopf-1 (DKK1), a secreted Wnt/β-catenin antagonist enriched in the BME. DKK1drives MDSC accumulation and immunosuppressive function by inhibiting β-catenin signaling in these cells [201]. Notably, DKK1 is preferentially expressed by tumors that metastasize to bone, where it suppresses osteoblast activity and promotes osteoclast-mediated resorption, an effect reinforced by the ability of MDSCs to differentiate into osteoclasts, together sustaining a pro-metastatic niche [202]. Thus, this DKK1-MDSC axis links immunosuppression, bone tropism, and bone remodeling, representing a promising therapeutic target in BoM.

## 11. Translational and Clinical Implications

### 11.1. Molecular Biomarkers in Bone Metastasis

In addition to what has been discussed previously, an expanded panel of BoM biomarkers spans multiple molecular classes: prognostic proteins including nPAK4, CAPG, GIPC1, DOCK4, serum RANKL, PRLR, and IL-1B; bone turnover markers P1NP, CTX, and uNTX; and circulating miRNAs including miR-19a, miR-16, and miR-378 [20,203]. In PCa, acquisition of an osteoblast-like phenotype, marked by OCN, ALP, BMP expression, Runx2 activation, and Wnt pathway engagement, serves as a tissue-based poor prognostic marker and a distinguisher between the osteoblastic PCa and other osteolytic cancers, while extracellular vesicle-derived miRNAs (miR-375, miR-21, miR-141-3p) isolated from blood, urine, and seminal fluid offer non-invasive assessment of bone-niche reprogramming before overt skeletal lesions are detectable [204]. In NSCLC, CXCR4 overexpression in areas of bone destruction promotes osteoclastogenesis via sVCAM1 secretion and ADAM17-mediated signaling, correlating with poor survival and enhanced osteoclastogenesis [205]. High SOST expression in primary BCa specimens independently predicts shorter bone metastasis-free survival and worse distant metastasis-free survival on multivariate analysis, identifying high-expressors as specifically susceptible to skeletal colonization and related events [206]. The MSC-MARCKSL1 subpopulation, enriched in primary tumors at the earliest MSC differentiation stage and capable of generating bone metastasis-specific downstream subpopulations, is proposed as a regulatory biomarker of early metastatic initiation [143].

### 11.2. Predicting Dormancy Versus Overt Progression

The dormancy mechanisms and reactivation triggers established in Section 5 and the molecular platforms described in Section 8 together provide the mechanistic and technological foundations for the following clinical biomarker strategies.

The CXCL5/CXCR2 axis executes the rate-limiting dormancy switch in bone: CXCL5 binding to CXCR2 is sufficient to awaken dormant tumor cells, while tumor cells simultaneously remodel the niche through enrichment of IL-1α, CXCL1, G-CSF, and IL-6, overcoming natural inhibitory signals partly by suppressing scavenger receptors ACKR1/DARC [207]. Complementarily, prolonged hypoxia downregulates LIFR in DTCs, triggering escape from hypoxia-induced dormancy and driving bone micrometastasis formation [41]. At the transcriptional level, MAD2L1 suppression, potentially identifiable through scRNA-seq of bone marrow aspirates, defines dormant tumor cells in bone-metastatic PCa, with high MAD2L1 expression independently associated with poor prognosis in TCGA cohorts [208]. The mTORC2/mTORC1 signaling balance provides a complementary clinically actionable biomarker: mTORC2 activity marked by RICTOR, pNDRG1, and Ki67-low/RBL2+ phenotype defines dormant bone marrow-resident cells and quiescent CTCs, while a shift to mTORC1-driven pP70S6K and Ki67/PCNA upregulation signals reactivation, both detectable via liquid biopsy or IHC to monitor CTCs for signs of early “asymptomatic progression” [209]. An elevated cfDNA-derived CIN score ≥12 independently predicts worse survival in bone-metastatic patients [210], while serial liquid biopsy, encompassing ctDNA and bone marrow aspirate-derived DTC profiling at the single-cell level, enables detection of dormancy escape up to 10.7 months before radiological progression [211].

### 11.3. Evolution-Informed Therapy

As established throughout this review, bone metastatic clones evolve under microenvironmental and therapeutic selective pressures rather than through primary tumor genetics alone. Effective intervention requires simultaneously targeting genetically defined clones, reversible epigenomic states, stromal dependencies, and dissemination capacity.

JAK inhibition overcomes BMSC-induced hormonal resistance mediated through IL-6/JAK/STAT3, while ERK and PI3K inhibitors address non-IL6-dependent compensatory bypass cascades, supporting the rationale for testing combination endocrine or androgen-axis therapy with pathway-matched inhibitors in molecularly selected patients [212]. Stromal dependencies converge on several combinable therapeutic axes: cytokine neutralization, targeting IL-11, IL-20RB, IL-1B, and G-CSF signaling, collectively dismantles osteoclastogenesis, pro-metastatic vascular remodeling, and the bone-tropic metastasis across breast and lung cancer models [46,188,189,213]; monocyte/macrophage-directed strategies including CCR2 and IL4R ablation alongside F4/80-targeted anti-CD137 nanoparticles further suppress macrophage-driven bone colonization [194,195]. Preclinical and early translational data support the rationale for combining checkpoint blockade with agents that relieve bone-niche immunosuppression, including IL-1β/TIGIT-directed strategies or RANKL-axis inhibition, although clinical benefit will likely depend on lesion type, tumor lineage, and immune ecosystem state [197,214]. Epigenomic plasticity is addressable through EZH2 inhibition, which prevents its capacity to seed other sites and reverses BME-induced ER silencing, restoring endocrine sensitivity [40,41], while SOST-STAT3 disruption by S6 and CXCR2 antagonism simultaneously targets bone-specific organotropism and the vicious cycle [206,207]. The targets discussed here serve as illustrative examples; additional therapeutic strategies relevant to BoM are addressed throughout the paper in their respective biological contexts.

### 11.4. Local Control Versus Systemic Evolutionary Control

The conventional framing of bone-directed therapy as purely palliative, aimed at preventing SRE, reflects an incomplete understanding of BoM biology that the evolutionary framework of this review fundamentally challenges. Bone is not a terminal destination but a dynamic launch pad: phylogenetic evidence demonstrates that visceral metastases across multiple organs are more closely related to bone metastatic lesions than to the primary tumor, positioning bone-to-organ seeding as a major driver of terminal multi-organ disease, as established in Section 7, meaning that an untreated bone lesion is an ongoing source of systemic evolutionary diversification rather than a locally confined problem [40,41]. The transition from purely palliative local intervention to integrated systemic strategies in spinal metastasis management reflects a clinical recognition, consistent with this evolutionary framework, that bone lesions are active participants in systemic disease progression rather than isolated endpoints [215]. Critically, bone lesions do not merely fail to respond to immunotherapy, they actively suppress ICI efficacy at extraosseous sites: osseous tumor-conditioned osteoclasts produce OPN that circulates systemically to reprogram distant tumor microenvironments and impair CD8+TCF1+ precursor T cell differentiation, with ICB responsiveness restored by αRANKL blockade or OPN neutralization and validated in clinical cohorts with the αRANKL-ICI combination regimen [216]. From a systemic evolutionary perspective, in oligometastatic PCa, the ORIOLE trial showed that SABR improved progression outcomes, and that total consolidation of PSMA-avid disease was associated with fewer new lesions at 6 months. Although not bone-specific, these findings support the broader principle that comprehensive treatment of visible metastatic reservoirs may influence subsequent dissemination patterns [217]. The inferior immunotherapy response rates in bone-metastatic patients, ORR 16% versus 41% in those without bone involvement, further underscore that bone lesions are not merely local problems but active modulators of systemic therapeutic resistance [218].

### 11.5. Clinical Trial Implications

Current BoM trials have been historically designed around skeletal-related event reduction, a narrow measure blind to the evolutionary and systemic dynamics established throughout this review. Addressing this gap requires both improved preclinical models and biologically informed trial stratification. The preclinical constraints described in Section 8 are directly relevant here: conventional PDX models are limited by species-specific barriers impeding human cell homing to rodent bone, while subcutaneous human bone implant models and osteoblast-collagen-hydroxyapatite matrix constructs in SCID mice better recapitulate the human bone niche, and have successfully elucidated roles of LIF, Wnt5a, and tumor-derived exosomes in dormancy and bone colonization, underpinning preclinical development of denosumab, bisphosphonates, and RTK inhibitors [219]. Iterative intracardiac injection of lung adenocarcinoma cells into mice, with selective harvest and re-injection of bone-homing subpopulations across four successive rounds, yields sublines with consistent and selective bone organotropism, providing a dedicated platform for studying the molecular pathways enriched in bone-adapted cells and evaluating therapeutic agents specifically against bone-metastatic disease [220]. At the biomarker level, a pan-cancer five-gene exosomal mRNA panel: HSP90AA1, SPP1, IL3, VEGFA, and PTK2, predicts BoM with >90% accuracy across breast, prostate, colon, and renal cancer, warranting prospective multi-center validation as early-detection biomarkers, therapeutic targets, and longitudinal monitoring tools in future BoM trials [221]. Critically, bone-only metastatic disease itself requires sub-stratification: de novo bone-only metastasis carries significantly inferior outcomes compared to relapse, while lytic lesion type, multiple bone sites, high tumor grade, and negative hormone receptor status each independently predict worse prognosis on multivariate analysis [222]. Future BoM trials must therefore stratify not only by bone-dominant versus visceral phenotype but also by biologically distinct subcategories within the skeletal compartment. Dormancy biomarkers, evolutionary signatures, immune ecosystem archetypes, and serial ctDNA dynamics should initially be incorporated as predefined translational or secondary endpoints, with the long-term goal of developing biologically informed primary endpoints.

## 12. Conclusions and Future Directions

BoM is best understood as a spatial evolutionary process in which tumor clones disseminate through selective bottlenecks, enter specialized skeletal niches, and undergo continued genetic, transcriptomic, epigenetic, and immune adaptation. This framework explains why bone lesions may remain clinically silent for years, why anatomically similar lesions can differ in therapeutic vulnerability, and why skeletal disease may influence systemic progression rather than simply represent an endpoint of metastatic spread.

Future progress will require integration of multiregion sequencing, single-cell profiling, spatial transcriptomics, liquid biopsy, and lineage-tracing models specifically optimized for mineralized tissue. The most important unresolved questions are whether bone lesions commonly reseed other organs, which dormant DTC states predict reactivation, and how bone-specific immune ecosystems determine response or resistance to systemic therapy. Addressing these questions will shift the field from descriptive BoM biology toward evolution-informed surveillance, trial design, and therapeutic intervention.

## Figures and Tables

**Table 1:** Comparative evolutionary and microenvironmental features of bone metastasis across breast cancer, prostate cancer, NSCLC, and ccRCC.

Feature	Breast Cancer	Prostate Cancer	NSCLC	ccRCC
Bone tropism	High (luminal-predominant)	Very high; near-universal in advanced disease	Moderate (30–40% incidence)	Moderate; distinct niche dependence
Timing of dissemination	Early dissemination in subset; context-dependent	Predominantly late dissemination	Often late; may involve intermediary metastases	Late dissemination with repeated seeding
Dormancy	Prolonged dormancy with late reactivation	Limited evidence for dormancy	Poorly characterized	Limited evidence
Clonal selection	Selection of reactivating subclones; endocrine-resistant lineages	Selection for AR-driven and osteoblast-adapted clones	Emerging evidence; oncogene-context dependent (EGFR, KRAS)	Selection for immune-evasive and stromal-adapted clones
Main bone phenotype	Predominantly osteolytic/mixed	Osteoblastic	Osteolytic	Osteolytic
Key adaptive pathways	WNT, EGFR, osteomimicry programs	AR signaling, WNT, BMP, endothelin-1	PTHrP, inflammatory cytokines; EGFR/KRAS contexts	RANK–RANKL, hypoxia, stromal remodeling pathways
Therapy-driven selection	ESR1 mutations; convergent endocrine resistance	AR amplification/mutation; shift to AR-independent/neuroendocrine states	Poorly characterized; limited data on resistance in bone	Limited evidence; immune escape predominates
Plasticity	High; luminal-to-basal/mesenchymal transitions	Moderate–high; lineage switching (NEPC)	Poorly defined	Moderate; EMT-associated programs
Spatial heterogeneity	High inter- and intra-lesional diversity; immune variation	Marked inter-lesional heterogeneity; regional intra-lesional variation	Emerging evidence of spatial/mechanical heterogeneity	High inter-lesional heterogeneity; clonally distinct lesions
Evolutionary pattern	Branched; reactivation of dormant clones	Parallel evolution across lesions	Branched evolution; multi-step seeding	Polyclonal seeding from soft-tissue reservoirs
Microenvironmental interaction	Niche-induced quiescence and reactivation; DTC reservoir	Osteoblast activation; dense niche construction	Osteoclast-driven “vicious cycle”; inflammatory milieu	Immune-excluded niche; TREM2^+^SPP1^+^ TAM–osteoclast axis
Genomic features	ESR1 mutations; subclonal diversification; epigenetic evolution	AR alterations; TP53/RB1 loss in AR-independent states	KRAS, EGFR; possible CDK4 amplification	SETD2, BAP1 mutations

## Data Availability

No new data were created or analyzed in this study. Data sharing is not applicable to this article.
